# Study Design and Lessons Learned From Using Google Takeout Donations for Suicide Prevention Research: Prospective Cohort Study

**DOI:** 10.2196/83637

**Published:** 2026-11-03

**Authors:** Katherine Anne Comtois, Payton Smythe, Amanda Kerbrat, Ethan H Kim, Natalie Crouch, Ava Homiar, Brittany Mosser, Nichole Sams, Michelle L Harvey, Courtney L Bagge, Trevor Cohen, Patricia A Arean

**Affiliations:** 1 Department of Psychiatry and Behavioral Sciences, School of Medicine, University of Washington, 325 9th Avenue, Box 359911, Seattle, WA, 98104, United States, 1 206-744-1752, 1 206-744-3231; 2 Krembil Centre for Neuroinformatics, Centre for Addiction and Mental Health, Toronto, ON, Canada; 3 Division of Clinical Informatics, Beth Israel Deaconess Medical Center, Boston, MA, United States; 4 School of Medicine, University of Washington, Seattle, WA, United States; 5 University of Michigan, Ann Arbor, MI, United States; 6 VA Center for Clinical Management Research, VA Ann Arbor Healthcare System, Ann Arbor, MI, United States; 7 Biomedical Informatics and Medical Education, School of Medicine, University of Washington, Seattle, WA, United States

**Keywords:** search engine data, digital clinical studies, remote clinical studies, suicide prevention, remote suicide prevention studies, Google data, Google Takeout

## Abstract

**Background:**

Social media and search engine tech companies, such as Google, use the wealth of data at their disposal to create algorithms to predict suicide risk and provide interventions based on company policies and outputs from these algorithms. Neither the details of these algorithms nor their accuracy in risk prediction are shared with the public; thus, research is needed to verify whether algorithms applied to search engine data can accurately predict suicide risk in a platform user. This paper provides new information about the methodological challenges in studying the value of search engine data for suicide prediction.

**Objective:**

This paper describes the study methods and experiences regarding recruiting and retaining participants in a year-long, fully remote prospective cohort study of suicide prediction in people with variable risk of suicide, using Google Search data.

**Methods:**

We aimed to recruit 1000 United States–dwelling and English-speaking adults with current suicidal ideation who fell into 1 of 3 categories: participants with a suicide attempt in the past year, participants with a lifetime suicide attempt but not one in the past year, and participants with no suicide attempt in their lifetime. Recruitment sources included online advertisements hosted on various websites, to differing levels of success. Participants completed online surveys and an interview via Zoom to enroll in the study and then were sent follow-up surveys every 2 weeks for the year following initial enrollment. Recruitment was conducted from March 2022 to January 2024, with follow-up data being collected through February 2025.

**Results:**

Of 15,120 records created in our Research Electronic Data Capture (REDCap) survey system, 485 participants were eligible to participate in the prospective year of the study. Recruitment challenges were mainly due to participant dropout across all enrollment time points, with 63.7% (9636/15,120) of the prescreening sample dropping out or refusing. Other issues included the difficulty of downloading Google Search data (446/3277, 13.6%) and not having enough data to meet the inclusion criteria among those who were able to successfully donate (252/3277, 7.7%). Lastly, the study encountered fraudulent behavior, with 489 of 15,120 records (3.2%) being identified as fraudulent over the course of the study.

**Conclusions:**

Despite past success with remote clinical research methods, we found that recruitment can be significantly hampered by fraudulent participation, despite the use of state-of-the-art methods to mitigate this problem. Further, the ability to acquire useful Google Search data is extremely challenging and necessitates the use of technical assistance and continuous monitoring of Google Takeout data structure to ensure accurate and complete data donations. The solutions we provide for this type of research should be helpful for future endeavors. However, research is still limited by the barriers to obtaining data from technology companies. These limitations have significant implications in detecting the accuracy and utility of suicide prediction from search engine data.

## Introduction

Approximately 4000 people died by suicide in the United States each month in 2023‐2024 [1]. Suicide is the second leading cause of death among youth aged 10 to 34 years, the fourth leading cause of death among individuals aged 35 to 44 years, and the fifth leading cause of death among those aged 45 to 54 years [2]. Between 2000 and 2021, the global age-standardized suicide rate dropped by 35% with the sole exception of the Americas, a region in which the rate rose by 17% [3]. Despite numerous attempts to refine the detection and prevention of suicidal thoughts and behavior, either through better diagnostic methods or the integration of detection and early prevention services in settings such as primary care medicine, the rates of suicidal thoughts and behavior in the United States continue to climb [4-19].

While our ability to identify individuals at increased risk is improving, our ability to determine when someone is at greatest risk is no better than chance, in part due to the low base rate of suicide and suicide attempts and the variable and fluctuating nature of suicidal ideation [20,21]. One type of data that has shown promise is social media use data (frequency and type of content viewed and posted) [5,13,22-29]. For instance, Bryan and colleagues [23,30] coded the public social media profiles of military personnel who died by suicide or other causes for the presence of stressful life situations (ie, triggers), somatic complaints or health issues (ie, physical problems), maladaptive or avoidant coping strategies (ie, behaviors), negative mood states (ie, emotions), and/or negative cognitive appraisals (ie, cognition) according to the fluid vulnerability theory. They found temporal sequences of content related to these domains that were unique to suicide vs death from other causes. Coppersmith and colleagues [22] were able to demonstrate that personal interactions with social media (eg, Facebook and Instagram) and data from smart devices such as phones and wearable devices could be used to infer a person’s mental health as well as ongoing risk of suicide [31-33]. However, a disadvantage of social media use data concerns the extent to which these data are representative of all individuals with suicide risk. Typically, social media users are young [34,35], with younger users posting more revealing and personal content than middle-aged and older users. In addition, because of data breaches and a mishandling of social media data, people are turning to locked groups where accessing posts is far more challenging [36-39].

Another similar source of continuous data is search engine history [40-44]. There are several reasons why individuals’ internet search history may be a more robust data source than social media for variable risk detection. Notably, 77% of the US population [45] seek information using search engines almost exclusively using Google Search [46]. The information gleaned from this source is less likely to be biased by the desire to display a positive social persona on social media [47]. This observation is consistent with prior research suggesting that conditions bearing social stigma are better represented in search log data than in social media posts [48]. Natural language processing and machine learning methods [6,49-51] that have been used to mine social media information may yield more accurate temporal suicide risk detection if applied to personal internet search data. The use of internet search data may prove to be an effective and immediate method of identifying warning signs and proximal suicide risk detection for anyone, regardless of their contact with systems of care or self-reported disclosures to other persons.

To this end, our team conducted pilot research to determine the utility of using Google Search data as warning signs for suicide attempt survivors [52]. A subset of participants with a prior suicide attempt enrolled in a clinical trial following a medical or psychiatric inpatient hospitalization were recruited for a substudy, led by the first author (KAC) [53], comparing internet search warning signs from Google Search data with their suicide data reported in study interviews. Sixty-two participants in the trial consented to be interviewed; of these, 47 (75.8%) agreed to share their private internet search data for this substudy, and 26 of those interviewed (26/47, 55.3%) were able to do so. Participants’ internet search behavior varied, with the median number of daily searches ranging from 1 to 25 over time periods ranging from 6 months to 6 years, with approximately 350,000 search queries provided in total. For some people, searches escalated and then stopped completely 1 day before a suicide attempt; for others, searches continued to escalate to a peak number of searches per day before a suicide attempt. Although these data were from a very small number of participants, they demonstrated the need to explore how search behavior could be used as an early warning sign for suicide risk.

Clearly, the potential of search engine histories to provide timely and actionable suicide prevention is high. However, to date, few have been able to successfully conduct research using these data outside the industry setting, and those within the industry rarely have data capturing if or when a suicide attempt or death by suicide has occurred. Search engine companies have been reticent about sharing their suicide risk algorithms, and, while they will collaborate with researchers on commercial devices (eg, smartwatch usage data), they very rarely collaborate with researchers using search engine data. Thus, to best understand the potential of search engine data to predict proximal risk for suicide—in other words, when someone will try to take their life—the onus rests on academics to develop methods to match data donations from search engine users with reports of suicidal thoughts and behavior.

This paper describes the methods we used to conduct a prospective, large-scale, and fully remote cohort study using Google Search data for predicting proximal risk for suicide in a national sample of people 18 years and older living in the United States, who were at variable risk for suicide. The aim of this paper is to describe the challenges faced in conducting a study of this nature. We discuss (1) sample representativeness, (2) challenges with screening out bots and people who are not who they claim to be (fraudulent actors), and (3) challenges with participant-driven data donations from commercial platforms such as Google Search.

## Methods

All study findings and discussion in this paper adhere to the Strengthening the Reporting of Observational Studies in Epidemiology (STROBE) checklist [54,55]. Our STROBE checklist is available in Checklist 1.

### Study Design

This prospective cohort study collected 1-year retrospective data via interview and prospective data via survey every 2 weeks for 1 year. To understand the flow of participants through study procedures, please see the steps of study enrollment for each participant in Figure 1. We planned to recruit a final sample of 1000 people based on previous online studies in which we were successful in recruiting large samples [56-58].

**Figure 1.**
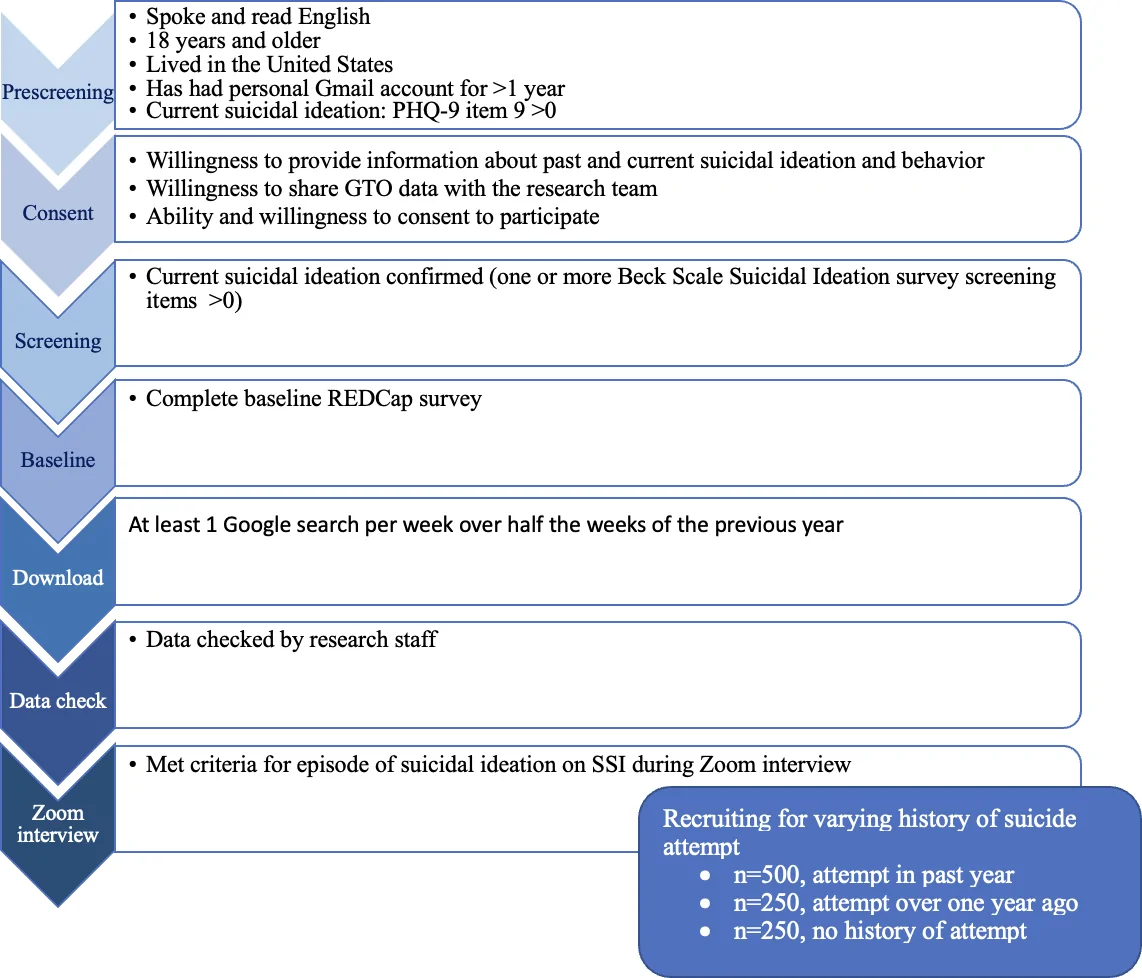
Steps of enrolling in the study and eligibility criteria corresponding to each step. Steps included prescreening, consent, screening, baseline, download, and Zoom interview. GTO: Google Takeout; PHQ-9: Patient Health Questionnaire; REDCap: Research Electronic Data Capture; SSI: Scale for Suicide Ideation.

#### Setting

All procedures were conducted online. Study surveys were administered online through Research Electronic Data Capture (REDCap; version 17.1.1; Vanderbilt University) [59,60]. The baseline interview was conducted via Zoom Workplace (version 6.7.7; Zoom Communications). Google data donations were conducted via Zoom and/or asynchronously through our web app. Recruitment for this study took place from March 2022 to January 2024, with follow-up data collection lasting until February 2025.

#### Ethical Considerations

The University of Washington Institutional Review Board (UW IRB STUDY00012455) approved the study protocol and all modifications. In addition to the UW IRB, a Data and Safety Monitoring Board (DSMB) was established, consisting of 3 researchers with relevant experience who were not affiliated with the study. We have obtained a Certificate of Confidentiality from the National Institutes of Health to protect participants’ identifiable data. Reporting to the UW IRB, DSMB, and funder conformed to protocol requirements. All data reported in this paper and in supplemental materials have been deidentified so that no identification of individual participants would be possible.

Informed consent was obtained from study participants using REDCap. A YouTube video detailing study procedures and the key elements of consent was presented to participants (see Multimedia Appendix 1 for a link to the video). After the participants reviewed the video and consent information, they were asked to complete a consent quiz to ensure that they understood the study protocol. Those who were unable to answer the quiz items correctly on the second attempt were presented with crisis and mental health resources and screened out of the study. After they correctly answered the consent questions, participants were given the choice to agree to participate in the study. The informed consent procedures had been thoroughly prepiloted and refined to maximize the understanding of the informed consent process and content. Participants were encouraged to contact the study team if they had any questions about the study or desired additional information. Participants had the opportunity to receive a total of US $240 via Amazon gift cards if they completed the entire study. Each study task had a different monetary incentive based on the degree of effort required of participants. Identifiable and deidentified data were collected from study participants. These data were stored separately, with a linking file with tightly limited access. Details of the study are documented in the informed consent documents in Multimedia Appendix 2.

#### Inclusion Criteria

Inclusion criteria for this study were (1) ability to speak and read English, (2) age 18 years or more, (3) place of residence in the United States, (4) a personal (not managed by employer or school) Gmail account held for at least 1 year, (5) current suicidal ideation (based on both survey and interview assessments), (6) willingness to provide information about past and current suicidal thoughts and behavior, (7) willingness to share Google Search data with the research team, and (8) ability and willingness to consent to participate. An additional and critical inclusion criterion was the ability to provide sufficient Google Search data at baseline, defined as at least 1 search per week for at least half the weeks in the past year. While YouTube searches were also downloaded as part of the study, the inclusion criterion for study aims was based exclusively on sufficient Google Search data. To ensure that a substantial portion of the sample had a history of a suicide attempt, an additional criterion of a history of suicide attempt was added during the study. This was intended to balance the sample such that 50% of participants would have made a suicide attempt in the past year and 25% would have made a lifetime suicide attempt (more than 1 year ago).

#### Recruitment Methods

Participants were recruited through online advertisements that provided a brief description of the purpose of the study, statement about potential incentives for completing study procedures, prescreening inclusion criteria, link to the study website, and contact information for the study team. Multiple online sources were used for recruitment including social media, Mental Health America’s website, ResearchMatch, the American Foundation for Suicide Prevention, and Prolific. Some participants also noted that they learned about the study from someone they knew. Thus, the sample was a volunteer convenience sample.

#### Assessments and Data Sources

Assessments used in this study are detailed in Table 1. For the purpose of this paper, the primary outcomes are descriptive. We report the following:

1. Number of participants sampled through each wave of recruitment and the demographics and location of the final sample
2. Proportion of recruited individuals who donated sufficient Google Search data, defined as at least 1 search per week for at least half the weeks over the past year
3. Number of fraudulent actors (people who claimed to be study participants but provided fraudulent information) we encountered and our fraud prevention procedures
4. Number of adverse events encountered
5. Retention of the sample at each assessment time point

Well-established self-report measures with strong psychometric properties were selected to measure 10 constructs shown to predict near-term risk for suicide attempt or were promising candidates. These were selected to compare the Google Search and YouTube findings against contemporaneous self-report as short-term (a period of weeks rather than hours or days) risk factors for attempt [61,62]. These measures were administered (all referencing a 2-week period) at all time points.

In addition to our primary measures, we assessed demographics and health service use. A survey at baseline asked about the participant’s use of search engines and social media as well as their comfort with and perceived helpfulness of possible internet search and social media suicide risk detection and intervention options [52,63].

**Table 1.** Summary of measures and data sources used.

Measure	At enrollment	Prospective (weeks 2‐50)	Week 52
Demographics and moderators			
Demographics	X	—^a^	X
Survey of technology use and technology-based help seeking	X	—	X
Service use	X	—	—
Interview assessment of suicidal thoughts and behavior			
Lifetime suicide attempts (SASI-Count^b^ interview)	X	—	—
Past-year suicide attempts (SASI-Count interview)	X	—	—
Suicidal ideation for past 2 weeks, worst in lifetime, and period of no suicidal ideation in past year, if any (Scale for Suicidal Ideation)	X	—	—
Self-report assessments			
Presence/absence of suicide attempt [64]	X	X	X
Suicidal ideation (Beck Scale for Suicide Ideation; BSS)	X	Items 1‐5	X
Balance between reasons for living and dying^c^ (BSS item 3)	X	X	X
Suicide-related communications^c^ (Suicide Attempt Self-Injury Interview) [65]	X	X	X
Preparation of affairs^c^ (BSS item 18)	X	X	X
Suicide method access and opportunity; preparations for a suicide attempt^d^ (BSS items 13 and 16)	X	X	X
Emotional experiences: empty^c^ (single item from Timeline Follow-Back Interview for Suicide Attempts [TLFB-SA] [46]), hostile, scared^c^ (Positive and Negative Affect Schedule—Expanded Form) [53,54]	X	X	—
Sleep difficulties^d^ (Insomnia Severity Index) [66]	X	X	X
Interpersonal negative life event^c^ (single item modified from the TLFB-SA) [61]	X	X	X
Alcohol use^c^ (2 items from the Alcohol Use Disorders Identification Test [AUDIT]: how often and, if any, how much) [67]	Lifetime and past 2 weeks	Past 2 weeks	Past year and past 2 weeks
Nonalcohol substance use^c^ (NIDA-Modified^e^ ASSIST)^f^ [68]	Lifetime version	Two items per substance: any use in past 2 weeks and, if any, how often	Past year only
Burdensomeness^c^ (single item from Interpersonal Needs Questionnaire) [69]	X	X	X
Google data			
Search + YouTube data	X	At 12, 24, and 36 weeks	X
Risk management			
University of Washington Revised Risk Assessment Protocol (UWRRAP)—online survey and interview versions	Online and interview	Online	Online

a Not applicable.

b SASI-Count: Suicide Attempt Self-Injury Count.

c These items measure constructs found to uniquely predict next-day suicide attempts in previous research: suicide-related communications, preparation of personal affairs, alcohol use, other central nervous system depressants, interpersonal negative life events, burdensomeness, absence of reasons for living, and the affective responses (feeling empty, hostile, and scared) [61,62].

d These items were included to assess potential short-term risk factors (suicide method preparation and sleep problems).

e NIDA: National Institute on Drug Abuse.

f ASSIST: Alcohol, Smoking and Substance Involvement Screening Test.

To illustrate the participant experience, we provide a summary of the clinical study measures in Table 1 and briefly describe the measures here. The Scale for Suicide Ideation (SSI) interview was used to determine episodes of current and worst suicidal ideation over the past year at baseline, whereas the self-report version, Beck Scale for Suicide Ideation (BSS), was used for the online surveys [70]. To reduce subject burden, only the first 5 screener items of the BSS were administered at the screening step and on prospective surveys. An item was added to the SSI to determine if and how long a participant experienced 1 or more periods of no suicidal ideation. Suicide attempts were assessed using the Suicide Attempt Self-Injury Count interview (SASI-Count) [65,71] to determine suicide attempts over the past year at baseline. In the online surveys, suicide attempts were captured by the question “Have you attempted to kill yourself?”, which has been demonstrated to identify a research-rated suicide attempt in 89.3% of cases with follow-up items regarding description, timing, and treatments received, if they reported an attempt [64].

The University of Washington Revised Risk Assessment Protocol (UWRRAP) was developed for research with participants with high and chronic suicidal ideation. This protocol has been used successfully in University of Washington Human Subjects Division–approved studies by multiple investigators in over 30 years of interview research (UWRRAP-Interview) [72-74]. During Zoom interviews, relevant resources were reviewed and provided afterward in a structured email. These included information about how to find help in a crisis, how to find mental health or other treatment, informal support options, lethal means counseling, coping skills, and other online resources. A version for entirely online research (UWRRAP-Online) was developed for data collected through REDCap. Survey items were designed to assess risk. On the basis of participant responses describing the past 2 weeks, resources to address suicide risk were provided. Resources were always shown in REDCap and depending on ratings of distress and suicidal desire “right now,” either the participants were asked if they would like a copy through email or text (when current distress and suicidal desire were low) or resources were automatically sent to the participant (when current distress or suicidal desire was high). REDCap sent these resources to their specified email or mobile phone number (whichever the participant preferred).

### Procedures

#### Prescreening, Consent, Screening, and Baseline Survey

Enrollment for each participant was conducted in several phases, which are illustrated in Figure 1 and described below. However, the study went through a series of methodological iterations to maximize participation. This is our focus here; thus, these are described in the Results section, and detailed information is provided in Multimedia Appendices 3 and 4. The procedures described below are the final procedures.

A study website provided information about the study and had a “Join Study” button that linked participants to the study’s REDCap survey system. The participants then completed the prescreening phase. Eligible individuals were asked to provide their first name, email, and mobile number to facilitate contact with the study team and were then routed to the consent form. Participants were informed about the 3 components of the study—surveys, interviews, and Google Search and YouTube data downloads. If they successfully completed the consent comprehension quiz and agreed to participate, they moved to the screening phase.

The screening phase included the first 5 screening items on the BSS. At this point, 3 additional questions were asked about the participants’ preferred search engine, preferred internet browser, and incognito mode usage to understand more about their search behavior. Eligible participants moved on to the baseline portion of the study (baseline survey, data donation, and baseline interview). If not, they were thanked for their time and interest and provided crisis and mental health resources.

#### Google Data

Google gives users access to their own data across their products, such as their Google Search history and product usage history (eg, Google Maps). This is then made available for download through an app called Google Takeout [75]. Data in this study consisted of retrospective and prospective Google and YouTube searches that participants were asked to share with the University of Washington investigators. We created an app called Google Takeout App (gTAP), to help participants donate their search and YouTube data without the need for them to share their Google credentials with the study staff. gTAP used Open Authorization 2.0 (OAuth 2.0), an industry-standard authorization protocol, to securely delegate access [76]. Participants accessed gTAP through a participant-specific and time point–specific link provided by a study team member during the interview appointment or shown to them through a REDCap survey (if the participant was completing the donation on their own). Participants completed all steps of the data donation on their own device and were instructed not to share any credentials with the study team member, even when being assisted by a team member. Once a participant initiated a Google data donation through the gTAP interface, Google Takeout prepared and delivered the data to the participant’s Google Drive (as it would for any Google user). gTAP then downloaded the data from their Google Drive using preapproved OAuth 2.0 encrypted credentials. The credentials were stored short-term (maximum 24 hours) to access participants’ Google Drive solely to locate the Google data file and allow the app to download the data (in most cases where the data files were not large, credentials were deleted within an hour of the download). The data were deidentified using Google’s Cloud Data Loss Prevention (DLP) application programming interface (API) to redact any sensitive search queries such as social security number and driver’s license number. Once the data were deidentified, gTAP downloaded the data into a secure cloud-based storage (Amazon Web Services S3) [77]. The videos used to instruct participants through the process can be found in Multimedia Appendix 1.

A backup method was created by the study team due to version control challenges between gTAP and Google Takeout. This method had participants download their Google data file to their own device (eg, phone, computer, or tablet) and upload it to REDCap. Participants were sent a personalized survey link to upload their data file. Once the study team received their data, the Google data were deidentified using the same DLP API as gTAP and stored as described above.

Those with insufficient Google data (download screening) or who did not meet the criteria for the worst-point episode of suicidal ideation in the past year on the Scale for Suicidal Ideation (Zoom interview) were thanked for their time and interest and provided the resources listed above. This ended their participation in this study. If the participant completed the Zoom interview and were screened in at both the download screening and Zoom interview steps, they were fully enrolled in the study and follow-up procedures.

#### Fraud Prevention Procedures

It is well-known that remote clinical research has a high risk of people joining a study for the purpose of achieving financial gain or some other malicious reason [78-81]. Given that participants received a financial incentive for participating (US $75 total at baseline), we were aware that we would need to monitor recruitment closely to screen out fraudulent actors. We used procedures reported in our other remote studies [56,57,82-88] and added further security by having participants complete a baseline interview over Zoom. Our process for preventing this issue evolved over time and resulted in a set of both automated and human-in-the-loop procedures detailed in Multimedia Appendix 5. If a participant was determined to be submitting fraudulent data, we flagged the participant as such in our REDCap database and discontinued their participation without pay.

#### Zoom Interview

Following the baseline survey, the participants were directed to an online appointment scheduling software to select an appointment for their Zoom interview with a study assessor. Interviews included the gold-standard retrospective assessments (SSI and SASI-Count) as well as UWRRAP risk management and generation of an individualized resource plan. If participants consented to having their interview recorded, we recorded audio and/or video based on their preference as stated in their consent form. Recordings were used to train study interviewers and for data quality assurance.

#### Risk Management and Resources

During the Zoom interview, interviewers cocreated a resource plan with participants using the UWRRAP-Interview version using the resource domains described above in the *Assessments and Data Sources* section. Participants were asked to share what supports or strategies they could use within each domain, and assessors would then offer online resources to participants to supplement their existing supports, as needed. A copy of the resource plan was then sent by email within 3 days of the interview. While the main purpose of the resource plan was to connect individuals to new resources, the information gathered in the process helped assessors evaluate suicide risk in cases where participants were distressed or reported suicidal urge or intent during the interview.

#### Engagement and Retention Strategies for Baseline Procedures

As detailed in Multimedia Appendices 3 and 4, we used a variety of strategies to improve initial retention, including sending study reminders through scheduling software at 7 days, 24 hours, and 1 hour before interview or data donation appointments, adding a mention of the Amazon gift code payment in email reminders, and reaching out to participants via their preferred contact method (email/text message) at the start of their appointments with an individualized Zoom meeting link. The Zoom meeting details and instructions on how to join the meeting were sent to participants when they initially scheduled their interview appointment as well as in the automated calendar reminders sent to them. To improve retention in baseline procedures, we also shortened the interview duration and increased the gift card values from US $20 to US $50 for the interview and from US $10 to US $25 for data donation.

#### Follow-Up: Surveys and Data Donations

Over the next year, enrolled participants received a text message or email (based on participant preference) every 2 weeks with a link to a brief prospective REDCap survey including the BSS screener, suicide attempt assessment for the past 2 weeks, and risk factor items. Each of the 26 prospective surveys had a 4-day window during which it could be completed before it expired. The final prospective survey at week 52 was longer and included all items of the baseline survey (including those in the prospective surveys) except the demographic questions not subject to change (eg, date or location of birth). During the prospective survey at weeks 12, 24, 36, and 52, participants were also prompted to donate their Google Search and YouTube data via a link to gTAP. Quarterly data donation was chosen because the most conservative deletion option for Google Takeout is 3 months, so this increased the chance of complete data through the follow-up year. If participants preferred to be walked through the data donation process by a study team member, they were provided with an automated calendar link to schedule a 15-minute Zoom appointment with a study team member.

### Data Analysis

To characterize the sample, frequency analysis was performed, and descriptive statistics were calculated for participant demographics. We also provide the number and percentage of participants who made it to each step in the enrollment process, as well as the reasons for which participants were excluded from the final sample. Percentages of prospective surveys completed were calculated and displayed in a bar chart.

## Results

### Sample Size Recruitment, Enrollment, and Retention

The CONSORT (Consolidated Standards of Reporting Trials) flowchart (Figure 2) describes how we obtained our final sample of 485 participants who met all eligibility criteria and completed the enrollment procedures with sufficient Google Search data. A summary of our iterations of enrollment procedures is described below. Specific details of each iteration and its results are provided in Multimedia Appendices 3 and 4, respectively.

At the start of the study (March 10, 2022), we asked participants to donate Google Search data on their own using gTAP. To maximize participation, we tried this both prior to and following the completion of the baseline survey. Of the participants in the download screening step asked to download their data on their own (first 4 procedure iterations, n=660), 76.7% (506/660) were lost because they could not donate data on their own (n=278) or lost to follow-up in the process (n=228). Because of the challenges participants reported while attempting to donate data on their own, we changed the procedures to have members of the study team guide participants through their Google data donation at the beginning of their Zoom interview (November 28, 2022). By providing real-time assistance, we lost only 88 participants in this iteration at baseline, mostly due to participant dropout. Although 1975 participants advanced to the download screening step, 325 participants could not donate Google Search data or had insufficient data, and 1063 failed to complete this assessment phase.

A review of potential fraud by research staff led to the exclusion of 272 participants due to 1 or more indicators of fraud. At the Zoom interview stage, 489 participants were eligible to participate based on all screenings. Four participants did not complete the interview, resulting in a final sample of 485 participants who met all inclusion criteria and completed all enrollment steps. Thus, we were not able to recruit 1000 participants as planned.

**Figure 2.**
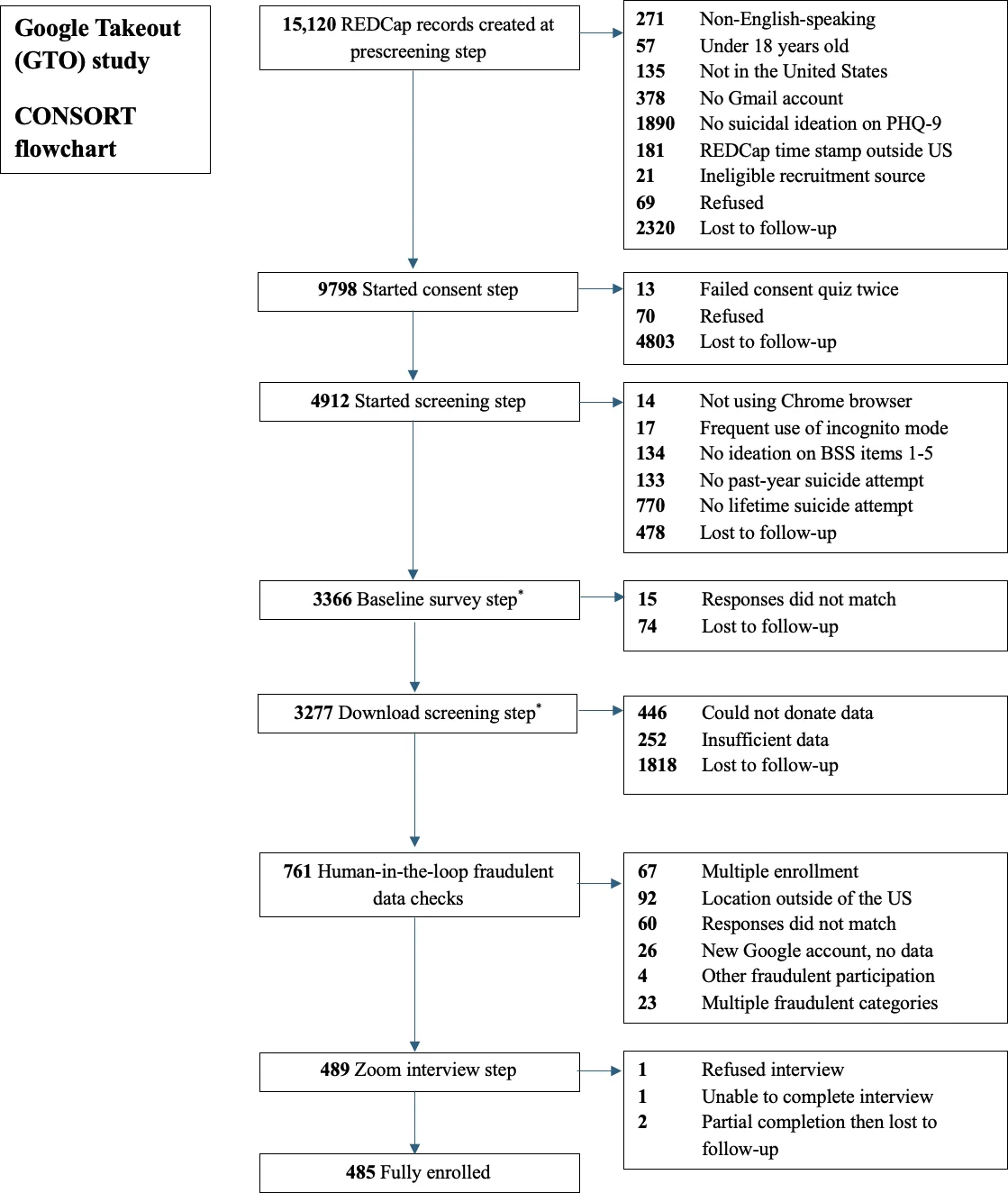
CONSORT flowchart displays the study enrollment flow and exclusion reasons. In total, 15,120 individuals entered the prescreening step, and 485 participants made it through all enrollment procedures and were eligible for the follow-up portion of the study. *In several study iterations (iterations 2-7 as detailed in Multimedia Appendices 3 and 4), the download screening step came before the baseline survey step—that is, only 2000 baseline surveys were completed, and not all 3277 individuals who entered the download screening step completed the baseline survey. BSS: Beck Scale for Suicide Ideation; CONSORT: Consolidated Standards of Reporting Trials; PHQ-9: Patient Health Questionnaire; REDCap: Research Electronic Data Capture; US: United States.

### Sample Demographics

Table 2 provides the characteristics of the final sample. The participants were 30.3 (SD 10.8) years on average and racially diverse, but there was a disproportionately higher percentage of women. More than half (268/485, 55.3%) of the participants identified as being LGBTQ+ (lesbian, gay, bisexual, transgender, queer, and other). We did not specifically attempt to oversample the LGBTQ+ community; however, we hypothesize that more people felt comfortable disclosing their sexual orientation and/or gender identity because our sample skewed younger and the study was conducted during a time when LGBTQ+ acceptance was high in the United States. Figure 3 displays the geographical representativeness of our sample, which included 47 of the 50 states plus Washington DC. This sample of 485 individuals, all of whom endorsed current suicidal ideation, included 95 (19.6%) participants who attempted suicide in the past year, 285 (58.8%) participants who attempted suicide more than a year ago, and 83 (17.1%) participants with no history of a suicide attempt.

**Table 2.** Demographic and clinical characteristics of the sample (N=485).

Variable	Value
Age (years), mean (SD)	30.3 (10.8)
Gender, n (%)	
Woman	331 (68.2)
Man	74 (15.3)
Nonbinary	32 (6.6)
Agender	4 (0.8)
Other gender	3 (0.6)
More than 1 gender	40 (8.2)
Missing data	1 (0.2)
Sexual orientation, n (%)	
Heterosexual or straight	203 (41.9)
Lesbian	22 (4.5)
Gay	9 (1.9)
Bisexual	108 (22.3)
Pansexual	61 (12.6)
Queer	37 (7.6)
Asexual	23 (4.7)
Not listed	6 (1.2)
Missing data	16 (3.3)
Transgender identity, n (%)	
No	401 (82.7)
Yes	70 (14.4)
Not listed	2 (0.4)
Missing data	12 (2.5)
Identifies as^a^ LGBTQ+^b^, n (%)	
No	199 (41)
Yes	268 (55.3)
Missing data	18 (3.7)
Marital status, n (%)	
Never married	298 (61.4)
Widowed	9 (1.9)
Married	116 (23.9)
Separated	18 (3.7)
Divorced	36 (7.4)
Missing data	8 (1.6)
Race and ethnicity, n (%)	
American Indian/Alaska Native	1 (0.2)
Asian	19 (3.9)
Black	32 (6.6)
Hispanic/Latino	22 (4.5)
Middle Eastern or North African (MENA)	0 (0)
Native Hawaiian or Other Pacific Islander	0 (0)
White	321 (66.2)
Other race	0 (0)
Multiracial	87 (17.9)
Missing data	3 (0.6)
Education, n (%)	
Did not graduate high school or obtain GED^c^	15 (3.1)
Graduated high school or obtained GED	84 (17.3)
Associate degree, technical training, or some college	227 (46.8)
Bachelor’s or graduate degree	158 (32.6)
Other	0 (0)
Missing data	1 (0.2)
Income source, n (%)	
Employment income only	244 (50.3)
Unemployment benefits only	6 (1.2)
Retirement income only	3 (0.6)
Disability benefits only	36 (7.4)
Retirement and disability benefits	1 (0.2)
Employment and other income	18 (3.7)
No source of income	159 (32.8)
Missing data	18 (3.7)
Income, n (%)	
Less than US $9000	105 (21.6)
US $9000 through US $44,999	243 (50.1)
US $45,000 through US $74,999	67 (13.8)
US $75,000 through US $99,999	27 (5.6)
US $100,000 or more	25 (5.2)
Missing data	18 (3.7)
Region in United States, n (%)	
Northeast	69 (14.2)
Midwest	106 (21.9)
South	169 (34.8)
West	141 (29.1)
Lifetime suicide attempt history^d^, n (%)	
Never	93 (19.2)
Single	99 (20.4)
Multiple	291 (60)
Missing data	2 (0.4)
Baseline survey suicide attempt classification^e^, n (%)	
No attempt (suicidal ideation only)	83 (17.1)
Attempt more than a year ago	285 (58.8)
Past-year attempt	95 (19.6)
Missing data	22 (4.5)
Baseline Suicidal Ideation (BSS), mean (SD)	14.6 (7.7)

a Based on gender, transgender, and sexual orientation variables.

b LGBTQ+: lesbian, gay, bisexual, transgender, queer, and other.

c GED: General Educational Development.

d Lifetime suicide attempt history was coded from the Suicide Attempt Self-Injury Count Interview (SASI-Count).

e Baseline survey suicide attempt classification was self-reported, which is why these values are slightly different than those for the same item assessed by an interviewer.

**Figure 3.**
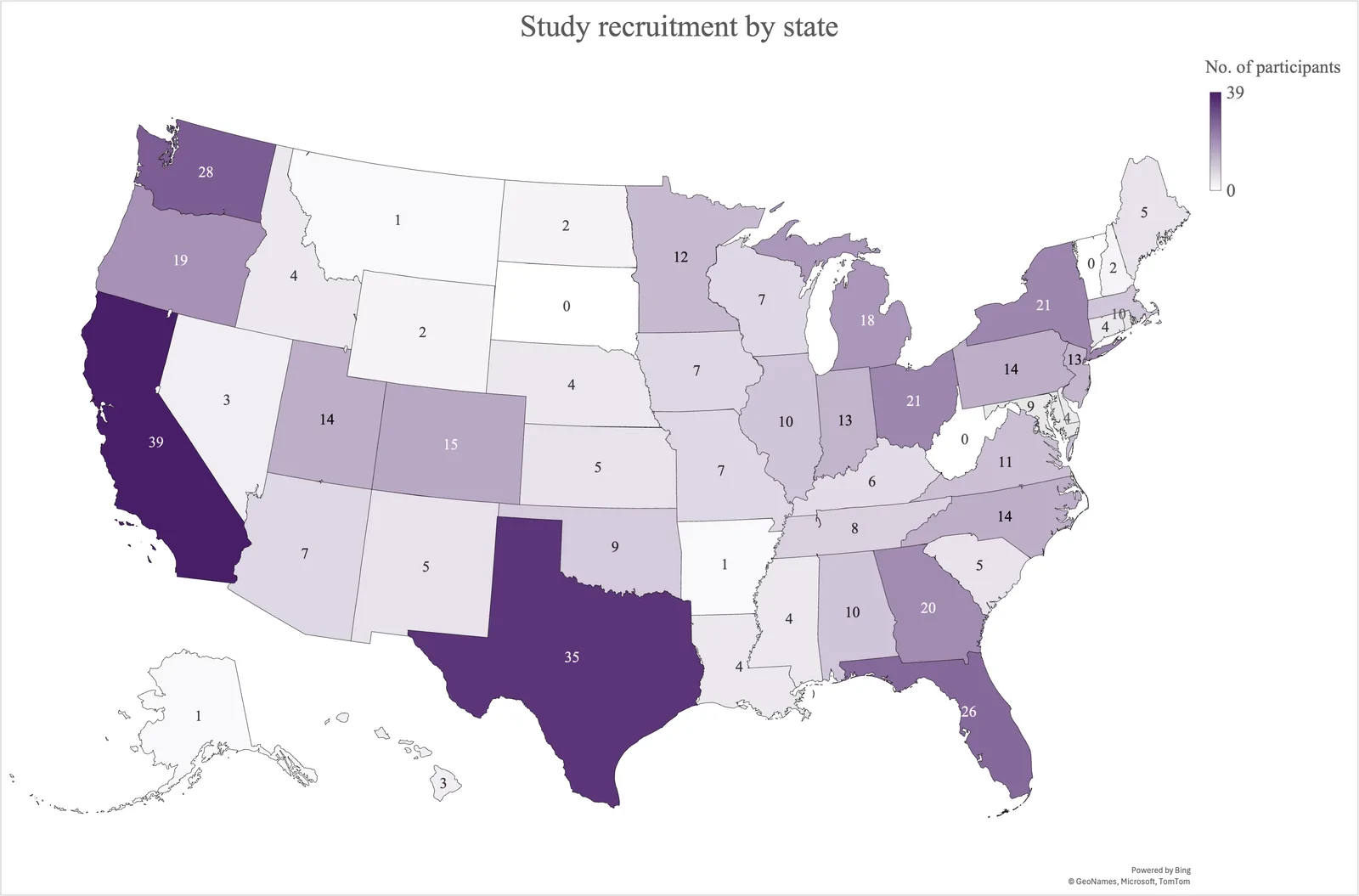
A map of the United States that displays the frequency of participants residing in each state at the time of study enrollment (created using the Bing Maps integration in Microsoft Excel [89], which is published under limited license per the Microsoft Bing Maps Terms of Use [90]).

### Retention of the Sample Through the Follow-Up Year

Participant attrition from baseline to week 52 was relatively low. As shown in Figure 4, the completion rates of the prospective surveys remained at 70% or higher across all time points. About three-quarters (71.8%, 348/485) of the fully enrolled participants successfully donated their Google data at week 52. Notably, 96.3% (335/485) of the participants who successfully donated their data had Google data spanning the full prospective year.

**Figure 4.**
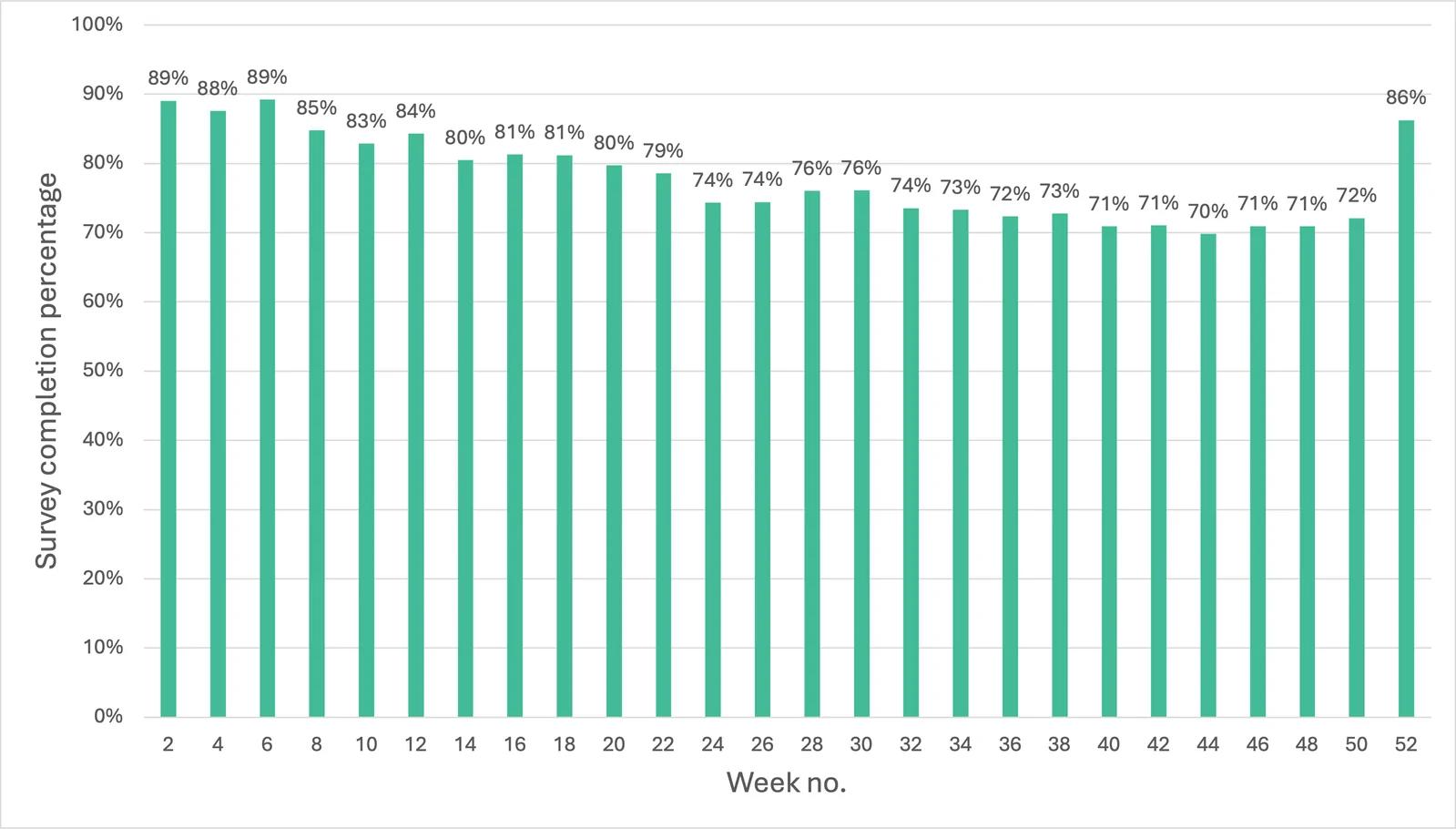
Percentage of prospective surveys completed by 485 enrolled participants at each prospective time point.

### Adverse Events During the Study

There were 124 suicide attempts (by 81 participants) reported during the prospective year. As suicide attempts were expected for our sample, they were not considered to be adverse events in this study by the IRB or DSMB. To our knowledge, 1 participant died, where the cause of death could not be determined (there being no indication that it was suicide). One participant threatened suicide if they were not enrolled in the study. This was addressed by the coprincipal investigator (KAC), a licensed clinical psychologist, who spoke with the participant and determined that they were in active treatment, had local resources available to them, and agreed to call 988, so no further intervention by the study was required.

### Recruitment Challenges: Dropouts, Google Takeout Availability, and Fraudulent Actors

A leading cause of our inability to meet our intended sample size was participant dropout at different points in the enrollment process. A total of 63.7% (9636/15,120) of the prescreening sample refused to provide consent or dropped out of the study, and 32.3% (4886/15,120) never consented (ie, refused, failed the consent quiz, or were lost to follow-up at this stage). There was less loss to follow-up during the screening (78/4912, 9.7%) and baseline survey phases (74/3366, 2.2%), and only 4 of the 489 individuals (0.8%) who completed the Zoom interview phase were lost to follow-up.

The second leading cause of recruitment attrition was a lack of sufficient Google Search data. Due to a difficulty with donating data, half of the participants (1818/3277, 55.5%) stopped interacting with the study. There were also issues with the download process, resulting in 13.6% (446/3277) of those in the download screening phase being unable to download their data. Moreover, 252 of the 3277 individuals (7.7%) were able to download their data but did not have sufficient searches to qualify for the study (ie, at least 1 Google Search per week for over half the weeks in the previous year).

The third reason for recruitment attrition was fraudulent actors. Of the 15,120 REDCap records at prescreening (the total initial sample), 489 (3.2%) were identified as fraudulent. Just under half (217/489, 44.4%) of the fraudulent actors was identified using automatic checks in REDCap, and the identification of the remainder required research staff time (272/489, 55.6%). The most prominent type of fraudulent acts found was that people were identified as being outside of the United States (273/489, 55.8%). Another common type of fraud was people enrolling in the study multiple times using the same contact information (67/489, 13.7%). Another commonly encountered type of fraudulent acts was identified when the responses given by a participant did not align with their previous responses (75/489, 15.3%).

## Discussion

### Methodological Lessons Learned

This study describes the methodological issues involved in conducting an entirely online study involving the download of Google Search data. Our goal was to enroll 1000 United States–dwelling and English-speaking adults experiencing suicidal thoughts or behaviors. The study was able to enroll 485 participants, and the final sample retention was high. The demographics of this sample were typical of remote, longitudinal studies of this nature [58]. We did encounter recruitment challenges owing to the need to screen fraudulent participation and valid participants experiencing difficulty downloading Google Search data or having insufficient amount of Google data to warrant participation. Despite our recruitment challenges, the final sample retention was high, and for participants who could provide data, the data quality was good; however, changes in policies at Google often interfered with the ability to download data. We discuss the specifics of these findings as lessons learned to share with other investigators who wish to pursue research of this nature [56,85,91-98].

### Lesson 1: Although Sample Representation Is Similar to Remote Studies, Recruiting Large Samples of Participants in a Search Engine Study Is More Challenging

Past remote clinical studies have demonstrated an ability to recruit very large samples that are nationally representative and have been able to quickly answer pertinent mental health questions [56,85,95,96]. Indeed, since COVID-19, the number of remote studies has grown considerably [97,98], including those on suicide research [91-93]. In this study, we demonstrated that it is possible to recruit a sample of people at varying risk for suicide from across the United States. Unlike previous remote trials [56,85,95,96], the ability to recruit a large sample, as was originally intended, was very challenging, and while many people attempted to enroll in our study, some were not who they said they were (lesson 2 below) or were unable to donate search data (lesson 3 below).

Our enrolled sample was much less prone to attrition during follow-up than other remote clinical studies [56,99], likely due to successful navigation of the baseline process, automated survey invitations in REDCap, a larger incentive (US $25 instead of US $5) for the longer final survey, and additional tracking and contacts by the study team to maximize completed data donations. However, this only represents 3% of the initial sample and 14% of those who passed screening on suicidality. As the most effective alternative of informing participants about the inclusion criteria prior to prescreening would have greatly increased the risk of fraud, future online studies will likely have to consider the impact of potential selection bias. We recommend that researchers work on methods to enroll and engage actual participants, as once a participant engages, the likelihood of continuing to participate for a year is high.

However, even with this sample size and retention rate, suicide attempts remained a rare outcome. Because of the relative sparsity of suicide attempts among participants, we anticipate that models developed to detect rare outcomes, such as anomaly detection techniques [100], will be most applicable to predicting attempts directly. However, on account of our high survey completion rate, it may also be possible to develop models of suicidal ideation and assess the extent to which predictions of severe ideation anticipate suicide attempts. In addition to sparsity at the outcome level, our data also exhibited sparsity at the feature level—the vast majority of internet searches did not concern content related to suicide. Natural language processing technologies present avenues through which to amplify informative signals by focusing modeling efforts on suicide-related searches [52].

### Lesson 2: The Cost of Screening Fraudulent Actors Is High, Regardless of Recruitment Strategy

It is well-known that fraudulent respondents and bots are a common problem with remote recruitment [78,80,101-106]. These have the potential to undermine data quality and result in several protocol changes to minimize the costs and burden of these respondents [105,107,108]. In this study, we used recruitment platforms that were noted, at the time, for their intensive screening procedures (eg, Prolific and ResearchMatch) and placed advertisements on advocacy websites (eg, Mental Health America and American Foundation for Suicide Prevention). Of these recruitment platforms, Mental Health America had the lowest percentage of fraudulent actors. To our surprise, ResearchMatch, which is a platform that is funded by the National Institutes of Health and is meant to help consumers find research studies for their condition(s), had the highest percentage of fraudulent actors before we started screening people out for endorsing ResearchMatch as their recruitment source. We are not alone in finding high fraudulent activity from ResearchMatch [107]. While Mental Health America was the most reliable and successful source for recruitment, the platform yielded a sample that skewed young and toward more women vs men or nonbinary people. We also flagged some behaviors as being suspicious (ie, using a virtual private network or inconsistent geolocation) that could have been nonmalicious [102,104]. While some people may have been excluded from the study, not providing this level of security leads to an influx of fraudulent participation [101,102]. Future researchers wishing to conduct remote recruitment may be best served using disease-specific advocacy websites and communities as sample sources. In another entirely remote study, the first author (KAC) recruited over 500 veterans in a year, with minimal fraudulent behavior, through a company focused on social media advertisement [109]. That being said, we are continuing to quantify the impact of fraud, including the monetary cost for fraud mitigation and identifiable differences between fraudulent responders and enrolled participants.

For our work, an important consequence of fraud mitigation is that our focus on an avenue of recruitment with a high yield for genuine participants constrained the demographics of our participant pool. In this context, cross-cutting models trained on the entirety of the pool may generalize poorly to participants from underrepresented demographic groups (eg, men). As the extent to which suicide-related search behavior may differ across these groups is not known, it will be important to compare model behavior across them at the point of validation. Should differences emerge, it may be desirable to develop subgroup-specific models. More broadly, it may be the case that the idiosyncrasies of search behavior favor individualized models over cross-cutting ones.

We recommend that for future research, samples may need to be enriched with certain populations that are less likely to engage in research focused on mental health concerns [110-113]. Open-source methods to detect fraudulent acts need to be developed and continuously updated as fraudulent actors adapt to security protocols [101,102,104,106]. For instance, if commercial tech companies could support scientists in the identification and recruitment of data donors who have legitimate corporate accounts and meet the inclusion criteria for location and available data, this assistance would lower the costs of conducting recruitment.

### Lesson 3: Data Donations Are Challenging

The inability to easily download Google Search and YouTube data was a challenge (see Multimedia Appendix 1 for an illustration of the download and sharing process). Of those who reached the download screening stage, over three-quarters were unable to download their data, had insufficient data, or did not complete this phase of the study. Over the course of the study, we pursued multiple options to make data donation possible. This included the creation of an app (gTAP) to help participants donate data directly to a secure cloud-based server, providing real-time assistance for donations, and combining data donations with the baseline interview. Google procedure for donation itself proved to be the biggest barrier to data donation. Google Takeout is a complicated tool with high user burden. Additionally, it is not uncommon for Google to change the data structure without warning, which required us to update gTAP several times [114]. Finally, Google made several policy changes about sharing search data during the course of the study such that a period of privacy review (2‐3 weeks per review) was necessary to approve gTAP. We also experienced intermittent failures of Google’s internal data redaction pipeline, which removes identifiable data from search logs. To process data collected during these periods, we developed an internal deidentification pipeline mirroring the redaction elements extracted by Google’s internal pipeline to ensure that future feature extraction methods could be consistently applied. Near the end of the study, we had to transition to a manual backup upload method due to yet another Google policy change. Using this data collection method reduced technical errors that were previously caused by the repeated challenge between Google and gTAP. Future research using Google Search history for disease prediction will need to factor in the many changes Google tends to implement around data donation. Additionally, investigators and funders should be aware of the potential costs associated with app updates beyond the typical costs for operating system updates and should budget for sustained technical support and updates accordingly.

Given these challenges, we recommend that open-source methods to facilitate data donation from search engines be developed to improve retention [115]. Search engine companies could help this process by creating constrained data sharing through standardized, secure APIs such that donors no longer need the technical expertise required to facilitate deposits themselves. Although Google provides a means for each individual to download their own data, the process is onerous for individuals and makes voluntary data sharing complex. Ideally, an individual should be able to provide permission for others to access their Takeout data easily, which is anticipated to both reduce the burden of technical support on research coordinators and improve recruitment rates.

### Limitations

This paper already details the many limitations associated with remote research to collect search engine data with individuals who consent to share their data—including representativeness of the sample and difficulties with obtaining Google data. Additional limitations of the study include the fact that it was conducted in the United States, and therefore participant behavior reported here will be more applicable to US participants. Some of the challenges regarding fraudulent acts reported in this paper may be due to the financial incentive offered for participation. Past studies have found that offering incentives increases the risk of fraudulent acts [101,116,117].

### Conclusion

This innovative study demonstrates the potential of fully remote studies to gather a national sample of participants with suicidal ideation beyond health care–based and school-based samples including both survey data and technology use data. We also elucidate the challenges and potential solutions to these challenges—particularly when merging data from technology companies who cannot or will not provide their data to researchers in batch form. Investigators who embark on studies of this nature will need to carefully consider their sources of recruitment, strategies to mitigate fraudulent actors, and the challenges in accessing search engine or other social media data and be realistic about the impact of these enrollment challenges on the generalizability of the sample recruited. We further conclude that providing capabilities for users to access their data is a necessary but inadequate level of support for research on risk detection using logs of internet search behavior. The systems currently in place are difficult to use for many participants and require additional development to facilitate sharing and integration of data for research purposes. Given the potential of these data to inform the prevention of serious events, companies such as Google have a moral imperative to work with scientists to develop algorithms for suicide risk prediction and mechanisms for secure data donation and to share their results with mental health researchers and/or practitioners while ensuring full transparency. Additionally, we call on these companies to work with external ethicists to improve the means by which they share—or do not share—data. Although there have been attempts to engage with these companies to provide external scrutiny of their own suicide risk algorithms [118], to date, these companies have declined. We recommend that scientists and technology companies collaborate in the validation of algorithms using corporate data within a protected enclave [119]. Until such partnerships grow, and technology companies facilitate donation, researchers will need to reserve resources for protection and adequate ongoing engineering support to oversee and correct for changes in search engine data management.

Prediction of proximal risk for suicide is the holy grail of suicide prevention. While we have a limited ability to determine who is at risk for suicide [120], current risk assessment only occurs when the person at risk interacts with school or health systems. Proximal risk for suicide remains elusive, as suicide risk fluctuates over time. We have demonstrated previously that search engine terms and behavior have the potential to both identify those at risk and potentially predict when an event will occur within a month of the event [52]. This ability to identify and intervene could substantially impact suicide rates around the world. To confirm this potential, large-scale research using these data is necessary. In this study, we have demonstrated that large-scale remote research is possible but not without its challenges and costs.

## Supplementary material

10.2196/83637 Multimedia Appendix 1 Consent and data donation instruction videos.

10.2196/83637 Multimedia Appendix 2 All study consent forms.

10.2196/83637 Multimedia Appendix 3 Iterations of enrollment procedures.

10.2196/83637 Multimedia Appendix 4 Participant status by iteration of enrollment procedure.

10.2196/83637 Multimedia Appendix 5 Detailed fraud prevention procedures.

10.2196/83637 Checklist 1 STROBE (Strengthening the Reporting of Observational Studies in Epidemiology) checklist.

## Abbreviations

API: application programming interface
BSS: Beck Scale for Suicide Ideation
CONSORT: Consolidated Standards of Reporting Trials
DLP API: Data Loss Prevention application programming interface
DSMB: Data and Safety Monitoring Board
gTAP: Google Takeout Application
GTO: Google Takeout
IRB: University of Washington Institutional Review Board
LGBTQ+: lesbian, gay, bisexual, transgender, queer, and other
OAuth: Open Authorization
REDCap: Research Electronic Data Capture
SASI-Count: Suicide Attempt Self-Injury Count
SSI: Scale for Suicide Ideation
STROBE: Strengthening the Reporting of Observational Studies in Epidemiology
UWRRAP: University of Washington Revised Risk Assessment Protocol
