# Supplementary material for: Study Design and Lessons Learned From Using Google Takeout Donations for Suicide Prevention Research: Prospective Cohort Study
Source: J Med Internet Res. 2026 Nov 3;28:e83637. doi: 10.2196/83637 (PMC13730293; doi:10.2196/83637)
Supplement: Supplementary file 1 [file jmir-v28-e83637-s002.pdf]

Approved
1/11/2023
UW IRB

UNIVERSITY OF WASHINGTON
CONSENT FORM
SEARCHLIGHT STUDY

Researchers:
Principal Investigator: Katherine Anne Comtois, PhD, MPH, Professor
Co-Principal Investigator: Patricia Areán, PhD, Professor
Department of Psychiatry and Behavioral Sciences/Harborview Medical Center
206-744-9353 searchlight@uw.edu

We are asking you to be in a research study. This form gives you information to help you decide whether or not to be in the study. Being in the study is voluntary. Please read this carefully. You may ask any questions about the study. Then you can decide whether or not you want to be in the study.

KEY INFORMATION ABOUT THIS STUDY

- We are asking if you would like to consent to a study—your participation is voluntary, and you do not have to agree.
- This study is looking at whether it is possible to learn about times of increased suicide risk based on Google Search and YouTube data. If so, we hope to develop online interventions to help at these critical times.
- If you have experienced suicidal thoughts, this study might be a good fit for you. If you tell us that you are at risk of suicide, we will offer you crisis and other mental health resources to help.
- To participate in the study, you will be asked to donate your Google Search and YouTube data, complete a phone or Zoom interview, and complete surveys about suicide attempts and/or thoughts of suicide that you experienced in the past year.
- Once you're in the study, we'll keep in touch for the next year asking you to complete brief surveys about any thoughts of suicide that you have experienced and donate your Google Search and YouTube data.
- You can earn up to $240 in Amazon gift codes by completing activities throughout the study. If you miss any study procedures, you will earn less than that amount.
- This is the first study to compare internet searches with times of high suicide risk, so we will also ask you for your opinions about whether and how we could help if increased risk can be detected.
- OPTIONAL: We will request your permission to obtain your medical records and to contact you for future studies, but these are not required to participate.
- You might **want** to participate in this study to help advance our understanding of how to help people who are suicidal or have made suicide attempts.
- You might **not want** to participate if you do not want to share your Google Search and YouTube data, do not want to meet with us for an interview, or do not want to complete surveys over the next year.

PURPOSE OF THE STUDY

This research study hopes to learn whether Google Search or YouTube data could be used to detect increased distress and risk of suicide in individuals who have experienced thoughts of suicide and/or have attempted suicide. In order to see if it is possible to predict your risk of suicide, we will compare your Google Search and YouTube data with times that you have and have not been thinking of suicide and the times that you attempted suicide, if you have. If it is possible to predict distress and suicide risk in this way, we plan to develop online interventions that can be offered to suicidal individuals at times when their risk is increasing.

No treatment is provided as part of this study and participating in this study is not a replacement for services provided by a qualified mental health professional. If you need immediate care to prevent harming yourself, please reach out to the following resources:

- National Suicide Prevention Lifeline at 1-800-273-8255 or chat online at https://suicidepreventionlifeline.org/chat/
- If you prefer to text, please contact the Crisis Text Line by texting HOME to 741741 or visit their website at https://www.crisistextline.org/

To explore getting help with mental health issues, including professional support, please visit Mental Health America:

- General information about finding and paying for mental health treatment as well as free wellness tools https://mhanational.org/finding-help
- Where to Get Help interactive tool for finding the best options for you: https://mhanational.org/get-involved/b4stage4-where-get-help-0

STUDY PROCEDURES

Participants in this study will be asked to complete the following baseline procedures:

Screening
After deciding whether or not you would like to continue in the study, we'll ask you to complete a few more screening questions (5 items). Once you complete these screening questions, we will let you know right away if it looks like the study is a good fit. Whether you are eligible for the study or not, we will give you some resources and contact information for immediate support.

Baseline Survey
If you are eligible to continue in the study, the first online survey will take 15-20 minutes and will ask about your mood, mental health, health care utilization, behaviors, attitudes, and opinions, including specifically asking about your experience with suicidal thoughts and any suicide attempts that you have made, if any.

You may refuse to answer any questions that you do not wish to answer in your surveys or interview. The most personal and sensitive questions we will ask are about whether you ever tried to kill yourself (and if so, what you did), what factors might be a trigger for your suicidal thinking, and your sexual orientation.

Baseline Interview

Approved
1/11/2023
UW IRB

After the baseline survey, we will ask you to schedule an interview with a member of our study team. This interview will take place via phone or Zoom, and we expect the interview will take 60-90 minutes.

At the beginning of the interview appointment, the study team member who will be interviewing you will help you to donate your Google Search and YouTube data. Anyone, regardless of study participation, can download their Google Search and YouTube data at any time. For the study, we will guide you through using a Searchlight web-app created by the study team called gTAP to donate your Google Search and YouTube data. In order to donate your data, you will give permission for the web-app to access and download your Google Search and YouTube data. As part of the data donation process, you will authorize Google to share your search data with the study team through the web-app. You will provide your Google credentials (i.e., username and password) directly on the Google website. The web-app will never access or store your Google credentials. The web-app will have temporary access to your Google Drive in order to securely download the data. The temporary access is revoked after download (usually immediately and always within 24 hours). Immediately after donating your Google Search and YouTube data, your data will be screened for the number of searches and number of weeks in the past year that you used Search and YouTube.

After you donate your data, the study team member will interview you to review your history of suicidal thinking and times you harmed yourself (if you have). We will also review the resources you have available to help with your suicidal thinking and mental health and add additional resources if we can. If you meet eligibility and complete the baseline survey and interview, you will receive a $50 Amazon gift code.

After the interview, we will know if your donated data was sufficient. If you do not have enough Google Search or YouTube data over the past year for the study analyses, you will not be eligible to continue in the study. If you have enough Google Search and YouTube data, you will be eligible to continue in the study and will receive an additional $25 Amazon gift code.

<u>Follow-Up Procedures</u>
Every two weeks over the next year, we will ask you to complete a short survey about your mood, suicidal thinking, and any attempts to harm yourself. These surveys are completed online and we will send you a text or email that includes a link when it is time for a survey. We expect that each of these surveys will take 3-10 minutes. For each survey that you complete, you will receive a $5 Amazon gift code (up to $110 total).

You will also be asked to donate your Google Search and YouTube data a few additional times over the next year. We will ask you to do this at 12 weeks, 24 weeks, and 36 weeks. For completing these data donations and the brief surveys at each of these timepoints, you will receive a $10 Amazon gift code (up to $30 total).

At the end of your year in the study, we will ask you to donate your Google Search and YouTube data one final time and complete a final survey. You will receive a $25 Amazon gift code for completing this final data donation and final survey. If you missed donating your Google Search and YouTube data at 12, 24, and/or 36 weeks, and your donation of Google Search and YouTube data at 12 months includes data from one or more of the missed periods, you will receive $5 for

Approved
1/11/2023
UW IRB

each quarter of data that your final donation fills in (between $5 and $15 in additional Amazon codes).

Some participants who are missing a large number of their follow-up surveys may be invited to do an interview at 12 months about when they experienced suicidal thoughts or made suicide attempts during the follow-up year. That interview would be like the baseline interview. If you are invited to complete a follow-up interview at 12 months, we will ask your consent for this at that time.

In case we are not able to reach you over the next year at the mobile number or email you provided, we ask your permission to reach out to you through contacts you provide to us during the interview. You will not be asked to provide contact information for anyone you do not want us to contact and no contact person you provide is obligated to share anything with the study.

The research team will review records with the Social Security Administration and National Death Index to determine overall mortality rates of participants enrolled in the study. Social Security Administration and National Death Index records review will occur approximately two years after your enrollment into the study with possible additional reviews five and ten years after that.

RISKS, STRESS, OR DISCOMFORT

Answering questions or talking about suicidal thoughts and behavior or triggers for suicidal thinking may be stressful to you. If you find this stressful, you are free to skip a question or stop at any time. Assistance from a crisis support line and other resources will be offered to you at the end of the survey and interview. If your responses suggest you might need immediate help, we will offer resources. At any time in the study, if you have concerns or the study team can provide additional resources or referrals, you can reach out to us.

As with all research, there is a chance that confidentiality might be compromised; however, we are taking precautions to minimize this risk.

BENEFITS OF THE STUDY

The purpose of this study is to see if it is possible to learn that someone is at risk of suicide from their Google Search and YouTube data. As this has never been studied before, we do not know if it will be possible. Therefore, we don't expect you will personally benefit from this study. What we learn from this study could help develop high-quality online interventions for people who are distressed or thinking of killing themselves so they can be helped before they harm themselves.

SOURCE OF FUNDING

The study is funded by the National Institute for Mental Health.

CONFIDENTIALITY OF RESEARCH INFORMATION

All information you provide us will be kept confidential with the following exceptions:

- If we have strong reason to believe that you are in danger of suicide, we will take steps

Approved
1/11/2023
UW IRB

to save your life. This may involve sharing information with clinicians or emergency services.
- If you tell us you are going to hurt a person you identified to us, we will connect you with someone who can help you prevent the danger and/or alert that person or the police.

Government or university staff (including institutional oversight review offices at the research site, the UW, or state and federal regulators) sometimes review studies such as this one to make sure they are being done safely and legally. If a review of this study takes place, your records may be examined. The reviewers will protect your privacy. The study records will not be used to put you at legal risk of harm.

We have a Certificate of Confidentiality from the National Institutes of Health. This helps us protect your privacy. The Certificate means that we do not have to give out information, documents, or samples that could identify you even if we are asked to by a court of law. We will use the Certificate to resist any demands for identifying information.

We can’t use the Certificate to withhold your research information if you give your written consent to give it to an insurer, employer, or other person. Also, you or a member of your family can share information about yourself or your part in this research if you wish.

There are some limits to this protection. We will voluntarily provide the information to:
- a member of the federal government who needs it in order to audit or evaluate the research;
- individuals at the institution(s) conducting the research, the funding agency, and other groups involved in the research, if they need the information to make sure the research is being done correctly;
- the federal Food and Drug Administration (FDA), if required by the FDA;
- individuals who want to conduct secondary research if allowed by federal regulations and according to your consent for future research use as described in this form;
- other authorities, if we learn of imminent intent to harm yourself or others requiring us to act to protect you or them.

The Certificate expires when the NIH funding for this study ends. Currently this is April 30, 2024. Any data collected after expiration is <u>not</u> protected as described above. Data collected prior to expiration will continue to be protected.

The information that we obtain from you for this study might be used for future studies. We may remove anything that might identify you from the information. If we do so, that information may then be used for future research studies, and/or given to another investigator without getting additional permission from you. It is also possible that in the future we may want to use or share study information that might identify you. If we do, a review board will decide whether or not we need to get additional permission from you.

OTHER INFORMATION

Approved
1/11/2023
UW IRB

You may refuse to participate and you are free to withdraw from this study at any time without penalty or loss of benefits to which you are otherwise entitled. If you wish to withdraw, please contact the researcher listed on page 1 of this consent form.

There is no cost to you for participating in this study. However, using the Searchlight web-app to donate your data and completing surveys on a phone or tablet may count against your mobile data plan. You may complete these procedures when your device is connected to WiFi to minimize your mobile data plan usage. The study will not provide any compensation or reimbursement for charges against your mobile plan.

You will receive Amazon gift codes for completing certain study procedures as described above and shown below. The maximum amount in gift codes for completing study procedures is $240.

1. Baseline survey and baseline interview	$50 Amazon gift code
2. Donation of sufficient Google Search data for the past year	$25 Amazon gift code
3. Brief follow-up surveys at two-week intervals (22 in all)	$5 Amazon gift code per survey (up to $110 total)
4. Brief follow-up survey and donation of Google Search and YouTube data at 12, 24, and 36 weeks (3 in all)	$10 Amazon gift code each time (up to $30 total)
5. 12 month survey and final donation of Google Search and YouTube data	$25 Amazon gift code

Amazon gift codes will be emailed to you. If you do not receive your gift code within five business days after completing a procedure, please contact the study at 206-744-9353 and we can troubleshoot so you receive the gift code within two weeks. If you choose to not complete a procedure or you choose to withdraw from the study once you have started, you will receive the reimbursement for the procedures you have completed by that time.

Because online research is vulnerable to fraud, multiple checks are employed to reduce fraudulent enrollment. Fraudulent participation will be discontinued without compensation.

If you would like a copy of this consent form, you may download a copy at the end of this screen, or one can be emailed to you at an email address that you provide. If you would prefer to receive a paper copy of the consent form at no cost to you, please contact the researchers listed on page 1 of this consent form.

RESEARCH-RELATED INJURY

If you think you have been harmed from being in this research or have any questions, complaints, or concerns about this study, you can contact Dr. Comtois and the research team at 206-744-9353.

QUESTIONS?

If you have any questions about this research study or this consent form, you may contact the research team at 206-744-9353 or searchlight@uw.edu.

Approved
1/11/2023
UW IRB

Because this is a research study, it is important that you know what is going to happen. Please answer these questions to confirm your knowledge.

If I am eligible, I will be asked to complete a survey today and every two weeks for one year, complete an interview about my suicide history, and permit the download of my Google Internet and YouTube data via Google's Takeout option. **TRUE**/FALSE

> TRUE: Correct! It's TRUE that if you are eligible, you will be asked to complete a survey today and every two weeks for the next year, complete an interview with a study assessor about your suicide history, and donate your Google Search and YouTube data to the study.
> FALSE: Oops, not quite! It's TRUE that if you are eligible, you will be asked to complete a survey today and every two weeks for the next year, complete an interview with a study assessor about your suicide history, and donate your Google Search and YouTube data to the study.

My participation is voluntary and I may exit the study without consequence. **TRUE**/FALSE

> TRUE: Correct! You can stop participating at any time. You will receive Amazon gift codes for the study procedures you completed. You can tell us to stop contacting you at any time and we will stop contacting you.
> FALSE: It is in fact TRUE that your participation is voluntary and you can stop participating any time you want. If you stop participating you will still receive Amazon gift codes for the study procedures you completed. You can tell us to stop contacting you at any time and we will stop contacting you.

If I meet eligibility requirements for this study, participation is for one year. **TRUE**/FALSE

> TRUE: Correct! If you are eligible, you will be in the study for one year.
> FALSE: Sorry, that is incorrect. It is TRUE that if you are eligible, your participation will be for one year.

I will receive Amazon gift codes based on how many procedures I complete. If I complete all study procedures, I will receive $240 in Amazon gift codes. If I miss surveys or data downloads, I will receive less than that amount. **TRUE**/FALSE

> TRUE: That's right! If you complete your baseline interview and every survey and all requested downloads of your Google Search and YouTube data, you'll receive a total of $240 in Amazon gift codes. If you miss any of your surveys or downloads of your Google Search and YouTube data, your total amount in gift codes will be lower.
> FALSE: That is actually true! We value your participation in the study and will send you gift codes for completing your interview, surveys, and downloads of your Google Search and YouTube data. You will receive up to $240 if you complete all procedures.

All the information I provide will be confidential, unless the study is being reviewed to make sure the research is being done correctly or if there is serious and immediate risk of harm to me or someone else. **TRUE**/FALSE

Approved
1/11/2023
UW IRB

TRUE: That's right! We will keep all of your answers private, unless agencies or groups involved in the research need the information to make sure the research is being done correctly, or if we believe you or someone else is in immediate danger.
FALSE: Nice try! It is TRUE that we will keep all of your answers private, unless the study is being reviewed by agencies or groups involved in the research if they need the information to make sure the research is being done correctly, or if we believe you or someone else is in immediate danger.

Thanks for answering our questions. Now is the time to let us know whether or not you would like to participate in the study. Please review the following statement:

This study has been explained to me. I volunteer to take part in this research. I have had a chance to ask questions. If I have questions later about the research, or if I have been harmed by participating in this study, I can contact one of the researchers listed on the first page of this consent form. If I have questions about my rights as a research subject, I can call the Human Subjects Division at (206) 543-0098 or call collect at (206) 221-5940. I can receive a copy of this consent form.

Now, please indicate if you consent to participate in the research:

☐ Yes, I agree to take part in this study.
☐ No, I do not want to take part in this study.

Approved
11/22/2022
UW IRB

Consent Language for Optional Study Procedures

We are now going to ask about some optional study procedures. The information in the main study consent form still applies.

(You were emailed a copy of the main consent form as soon as you agreed to participate. Please email searchlight@uw.edu if you didn't get it or would like another copy.)

CONTACT FOR FUTURE STUDIES

We would also like your permission to contact you about additional interviews or an additional follow-up study. Saying yes now does not mean you have to do the additional interviews. Your permission simply allows us to contact you.

☐ Yes, I agree to be contacted by the study team about additional interviews or studies.

☐ No, I do not agree to be contacted by the study team about additional interviews or studies.

REVIEW SERVICE UTILIZATION DATA

We would like to review data on your use of emergency room and hospitalizations for the five years prior to your enrollment and five and ten years after your enrollment. This would allow us to see if Google Search and YouTube data predict your use of these services. This is optional and you do not have to agree in order to participate in the rest of the study.

☐ Yes, I give permission for the study to review data on my use of emergency room and hospitalizations for the five years prior to today, and five to ten years after today.

☐ No, I do not give permission for the study to review data on my use of emergency room and hospitalizations for the five years prior to today, and five to ten years after today.

INTERVIEW RECORDINGS

We would like to video or audio record the interview part of the study that you complete with a member of the study team for training and quality assurance purposes. The recording is completely voluntary. You may continue participation in the study whether you agree to be recorded or not.

Recordings are labeled with a code and not with your name, and are kept on a password-protected secure server separate from your other study information. If you agree to have an interview recorded for quality assurance purposes, the recording will be destroyed at the end of the study and after records retention requirements have been met.

Please select:

☐ Yes, I give my permission for the interview to be recorded.

- ☐ I give permission for video as well as audio recording
- ☐ I give permission for audio recording only

☐ No, I do not give my permission for the interview to be recorded.

Approved
11/22/2022
UW IRB

NATIONAL INSTITUTE OF MENTAL HEALTH DATA ARCHIVE

Data from this study may be submitted to the National Institute of Mental Health Data Archive (NDA). NDA is a data repository run by the National Institute of Mental Health (NIMH) that allows researchers studying suicide to collect and share de-identified information with each other. A data repository is a large database where information from many studies is stored and managed. We will collect information from you to create a unique ID code that cannot be linked to your identity. This information includes your legal name at birth, your date of birth, gender assigned at birth, and city where you were born. De-identified information means that all personal information about research participants such as name, address, and phone number is removed and replaced with a code number. With an easier way to share, researchers hope to learn new and important things about suicide more quickly than before.

During and after the study, the researchers will send de-identified information about your health and behavior to NDA. We will not share your Google Search and YouTube data. Other researchers nationwide can then file an application with the NIMH to obtain access to your de-identified study data for research purposes. Experts at the NIMH who know how to protect health and science information will look at every request carefully to minimize risks to your privacy.

You may not benefit directly from allowing your information to be shared with NDA. The information provided to NDA may help researchers around the world to treat people at risk of suicide so that they have better outcomes. NIMH will also report to Congress and on its website about the different studies that researchers are conducting using NDA data. However, you will not be contacted directly about the data you contributed to NDA.

You may decide now or later that you do not want to share your information using NDA. If so, contact the researchers who conducted this study, and they will tell NDA, which can stop sharing the research information. However, NDA cannot take back information that was shared before you changed your mind. If you would like more information about NDA, this is available online at https://nda.nih.gov

☐ **I wish to share my information using NDA** .

☐ **I do not want to share my information using NDA**.

I have had a chance to ask questions. If I have questions later about the research, or if I have been harmed by participating in this study, I can contact one of the researchers listed on the first page of the main consent form or by emailing searchlight@uw.edu.

Consent Language for Optional Manual Google Data Upload

As you have experienced, our Searchlight web-app has been experiencing unfixable technical errors when participants have attempted to share their Google Search and YouTube data. Based on that, we are asking you to consider an optional study procedure. The information in the main study consent form still applies.

(You were emailed a copy of the main consent form as soon as you agreed to participate. Please email searchlight@uw.edu if you didn't get it or would like another copy.)

GOOGLE DATA UPLOAD TO REDCAP

We would also like your permission to receive your Google Search and YouTube data from our secure survey system, REDCap. This is the same secure system where you have been filling out your online surveys. To do this, you will upload your exported Takeout file to REDCap directly, instead of going through our Searchlight web-app. If you prefer not to do this, we will not collect your Google Search and YouTube data this way.

☐ Yes, I agree to upload my Google Search and YouTube data to REDCap.

☐ No, I do not agree to upload my Google Search and YouTube data to REDCap.

I have had a chance to ask questions. If I have questions later about the research, or if I have been harmed by participating in this study, I can contact one of the researchers listed on the first page of the main consent form or by emailing searchlight@uw.edu.
