# Supplementary material for: Study Design and Lessons Learned From Using Google Takeout Donations for Suicide Prevention Research: Prospective Cohort Study
Source: J Med Internet Res. 2026 Nov 3;28:e83637. doi: 10.2196/83637 (PMC13730293; doi:10.2196/83637)
Supplement: Supplementary file 2 [file jmir-v28-e83637-s001.docx]

Multimedia Appendix 1. Consent and data donation instruction videos.

Consent Video: <https://www.youtube.com/watch?v=36KxSLUkl9Q&t=62s>

Part 1 - Web App Authorization: <https://www.youtube.com/watch?v=PGJQWUVaioI>

Part 2 - Data Donation: <https://www.youtube.com/watch?v=9_GfzakBMQY>
