# Supplementary material for: Study Design and Lessons Learned From Using Google Takeout Donations for Suicide Prevention Research: Prospective Cohort Study
Source: J Med Internet Res. 2026 Nov 3;28:e83637. doi: 10.2196/83637 (PMC13730293; doi:10.2196/83637)
Supplement: Supplementary file 3 [file jmir-v28-e83637-s003.docx]

Multimedia Appendix 3. Iterations of enrollment procedures.

| Iteration | Description | n (%) |
| --- | --- | --- |
| 1 | Pre-Screening à Screening (includes search behavior screen) à Baseline Survey à Download Screening on their own ($10) àInterview ($30) | 143 (0.9%) |
| 2 | Pre-Screening à Screening (includes search behavior screen) à Download Screening on their ownà Baseline Survey ($10) àInterview ($30) | 221 (1.5%) |
| 3 | Pre-Screening à Screening à Download Screening on their own à Baseline Survey ($10) àInterview ($30) | 209 (1.4%) |
| 4 | Pre-Screening à Screening à Download Screening on their own à Baseline Survey à Interview ($40) | 1,440 (9.5%) |
| 5 | Pre-Screening à Screening à Download Screening scheduled 1:1 appt to donate à Baseline Survey à Interview ($40) | 536 (3.5%) |
| 6 | Pre-Screening à Screening à Download Screening drop-in 1:1 appt to donate à Baseline Survey à Interview ($40) | 705  (4.7%) |
| 7 | Pre-Screening à Screening à Download Screening drop-in appt or on their own à Baseline Survey à Interview ($40) | 1,136 (7.5%) |
| 8 | Pre-Screening à Screening à Baseline Survey à Download Screening and Interview ($20) à Sufficient data confirmed ($10) | 1,384 (9.2%) |
| 9 | Pre-Screening à Screening à Baseline Survey à Download Screening and Interview ($50) à Sufficient data confirmed ($25) | 1,294 (8.6%) |
| 10 | Pre-Screening à Screening (past year suicide attempt only) à Baseline Survey à Download Screening and Interview ($50) à Sufficient data confirmed ($25) | 689 (4.6%) |
| 11 | Pre-Screening à Screening (past year suicide attempt only) à Baseline Survey à Download Screening and Interview (interview shortened) ($50) à Sufficient data confirmed ($25) | 434 (2.9%) |
| 12 | Pre-Screening à Screening (lifetime suicide attempt only) à Baseline Survey à Download Screening and Interview (interview shortened) ($50) à Sufficient data confirmed ($25) | 6,929 (45.8%) |
