# Supplementary material for: Study Design and Lessons Learned From Using Google Takeout Donations for Suicide Prevention Research: Prospective Cohort Study
Source: J Med Internet Res. 2026 Nov 3;28:e83637. doi: 10.2196/83637 (PMC13730293; doi:10.2196/83637)
Supplement: Supplementary file 4 [file jmir-v28-e83637-s004.docx]

Multimedia Appendix 4. Participant status by iteration of enrollment procedure.

|  | **Procedure Iteration** | | | | | | | | | | | |
| --- | --- | --- | --- | --- | --- | --- | --- | --- | --- | --- | --- | --- |
| **Status** | **1** | **2** | **3** | **4** | **5** | **6** | **7** | **8** | **9** | **10** | **11** | **12** |
| Fully enrolled | 1 | 2 | 6 | 23 | 13 | 5 | 16 | 59 | 70 | 3 | 10 | 277 |
| Pre-Screen out: Non-English speaking | 3 | 13 | 9 | 15 | 6 | 13 | 14 | 27 | 25 | 11 | 8 | 127 |
| Pre-Screen out: Under 18 years old | 0 | 3 | 3 | 4 | 1 | 0 | 9 | 3 | 4 | 4 | 0 | 26 |
| Pre-Screen out: Endorsed not in the United States | 4 | 2 | 0 | 6 | 0 | 2 | 7 | 5 | 5 | 2 | 0 | 102 |
| Pre-Screen out: No Gmail account | 0 | 4 | 6 | 36 | 12 | 16 | 38 | 28 | 35 | 18 | 11 | 174 |
| Pre-Screen out: No suicidal ideation on PhQ-9 | 13 | 21 | 31 | 157 | 75 | 100 | 119 | 156 | 172 | 76 | 43 | 927 |
| Pre-Screen bad actor: REDCap timestamp outside of US | 0 | 0 | 0 | 8 | 1 | 3 | 8 | 11 | 11 | 3 | 3 | 133 |
| Pre-Screen bad actor: ineligible recruitment source | 0 | 0 | 0 | 0 | 0 | 0 | 0 | 0 | 0 | 0 | 0 | 21 |
| Pre-Screen refused | 1 | 0 | 2 | 3 | 1 | 2 | 3 | 12 | 4 | 4 | 2 | 35 |
| Pre-Screen lost to follow-up | 10 | 30 | 33 | 260 | 92 | 109 | 194 | 265 | 247 | 121 | 75 | 884 |
| Consent quiz failure | 1 | 0 | 0 | 2 | 0 | 0 | 1 | 1 | 1 | 1 | 0 | 6 |
| Consent refused | 0 | 0 | 0 | 8 | 3 | 1 | 4 | 7 | 7 | 7 | 3 | 30 |
| Consent lost to follow-up | 26 | 35 | 71 | 450 | 195 | 254 | 388 | 425 | 419 | 230 | 147 | 2163 |
| Screen out: Not using Chrome browser | 4 | 10 | 0 | 0 | 0 | 0 | 0 | 0 | 0 | 0 | 0 | 0 |
| Screen out: Frequent use of incognito | 10 | 7 | 0 | 0 | 0 | 0 | 0 | 0 | 0 | 0 | 0 | 0 |
| Screen out: BSS items 1-5 all zero | 2 | 0 | 0 | 11 | 6 | 3 | 10 | 17 | 4 | 8 | 4 | 69 |
| Screen out: No past year suicide attempt | 0 | 0 | 0 | 0 | 0 | 0 | 0 | 0 | 0 | 78 | 55 | 0 |
| Screen out: No lifetime suicide attempt | 0 | 0 | 0 | 0 | 0 | 0 | 0 | 0 | 0 | 0 | 0 | 770 |
| Screen lost to follow-up | 2 | 0 | 1 | 3 | 0 | 1 | 9 | 78 | 52 | 101 | 43 | 188 |
| Baseline Survey bad actor: non lifetime SA/responses not matching T2screen | 0 | 0 | 0 | 0 | 0 | 0 | 0 | 0 | 0 | 0 | 0 | 15 |
| Baseline Survey lost to follow-up | 1 | 0 | 0 | 0 | 0 | 0 | 1 | 8 | 13 | 2 | 1 | 48 |
| Download Screening: could not donate data | 18 | 8 | 14 | 238 | 8 | 3 | 53 | 15 | 12 | 1 | 1 | 75 |
| Download Screening: insufficient data | 8 | 1 | 1 | 20 | 8 | 1 | 18 | 33 | 28 | 1 | 1 | 132 |
| Download Screening lost to follow-up | 9 | 7 | 30 | 182 | 114 | 191 | 231 | 226 | 177 | 17 | 19 | 615 |
| Bad actor: Multiple enrollments | 14 | 5 | 2 | 9 | 0 | 0 | 8 | 1 | 0 | 0 | 6 | 22 |
| Bad actor: Location outside of United States | 3 | 44 | 0 | 2 | 1 | 1 | 0 | 3 | 2 | 0 | 2 | 34 |
| Bad actor: Responses don’t match | 0 | 2 | 0 | 2 | 0 | 0 | 3 | 1 | 2 | 1 | 0 | 49 |
| Bad actor: New Google Account, no data | 12 | 5 | 0 | 1 | 0 | 0 | 1 | 2 | 2 | 0 | 0 | 3 |
| Bad actor: Other | 0 | 0 | 0 | 0 | 0 | 0 | 0 | 1 | 0 | 0 | 0 | 3 |
| Bad actor: Multiple fraud categories | 1 | 22 | 0 | 0 | 0 | 0 | 0 | 0 | 0 | 0 | 0 | 0 |
| Interview: Refused | 0 | 0 | 0 | 0 | 0 | 0 | 1 | 0 | 0 | 0 | 0 | 0 |
| Interview: Unable to complete | 0 | 0 | 0 | 0 | 0 | 0 | 0 | 0 | 1 | 0 | 0 | 0 |
| Interview lost, after partial | 0 | 0 | 0 | 0 | 0 | 0 | 0 | 0 | 1 | 0 | 0 | 1 |

Columns: Enrollment Procedures as shown in Multimedia Appendix 1.

Rows: Reasons why enrolled or excluded, refused, or lost-to-follow-up as shown in the Figure 2 CONSORT flow chart.
