# Supplementary material for: Study Design and Lessons Learned From Using Google Takeout Donations for Suicide Prevention Research: Prospective Cohort Study
Source: J Med Internet Res. 2026 Nov 3;28:e83637. doi: 10.2196/83637 (PMC13730293; doi:10.2196/83637)
Supplement: Supplementary file 5 [file jmir-v28-e83637-s005.docx]

Multimedia Appendix 5. Detailed Fraud Prevention Procedures

1. Timestamp capture: We included a REDCap field to capture the participant’s device timestamp and calculated the difference between a participant’s device timestamp and Pacific time. Participants with a device timestamp greater than 3.05 hours difference were automatically screened out, as that indicated that the participant’s device was in a non-United States time zone.
2. Information verification: Our screening surveys included items assessing the same concept at different timepoints during the screening process (see Figure 1 for flowchart of the study process). An item asking participants for their zip code was added to the Pre-Screening phase and an item asking participants to type their city and state was added to the Screening phase. Study team members would compare a participant’s zip code, city and state, and device time zone to make sure that they aligned. If they did not agree, participants were deemed fraudulent and were discontinued from the study.
3. Verification of recruitment sources: Because ResearchMatch, a research recruitment platform, resulted in a high number of fraudulent actors, we added an item to the beginning of our pre-screen asking participants where they found the study and screened out people who selected ResearchMatch as their recruitment source.
4. Zoom location: As our inclusion criteria was United States residents, we prevented potential participants who appeared to be from outside of the United States from joining the Zoom baseline interview using Zoom’s internal settings.
5. Google Takeout App time stamp: We also enabled gTAP to block participants from donating their Google data if they were located outside of the United States and Canada (Canada was not blocked due to some of the gTAP developers residing in Canada).
6. Google Search data completeness: Participants donating Google data from an account created 14 days ago or less were screened out and were flagged by the study team as fraudulent. This did not include good faith participants that had the search tracking feature on their Google account turned off (as many participants did), which could be differentiated by a specific gTAP error.
7. Other procedures: Indicators that were deemed as fraudulent included having an IP address located outside of the United States, multiple participants donating data from the same Google account, and duplicate enrollments that have substantially differing survey responses (rather than someone enrolling multiple times due to their issues with the survey where their responses were comparable).
8. Indicators that were deemed as suspicious (not being enough to determine data was fraudulent on its own, but in combination with other suspicious indicators could rule the data as fraudulent): included using a Virtual Private Network (VPN) in the interview, reporting no self-injury in the baseline interview in contradiction to their survey data, and having issues confirming their contact information during the baseline interview.

Our consent form included language specifying that behavior deemed fraudulent by the research team would be discontinued: “Because online research is vulnerable to fraud, multiple checks are employed to reduce fraudulent enrollment. Fraudulent participation will be discontinued without compensation.” If we needed to let them know why their interview appointment was canceled so they would not schedule a new one, we would send them this message: “Hi Name, your study record was flagged for fraudulent responses. You are not eligible to continue in the study, and your appointment has been cancelled. -Searchlight Study"

Staffing for the study consisted of 0.5 full time equivalent staff focused on the website and recruitment methods and 3-4 full time equivalent staff focused on data collection of consent, baseline surveys, baseline interviews, fraud prevention, and prospective follow-up surveys. Technology staff designed (0.5 full time equivalent) and maintained (0.1 full time equivalent) the web application for Google data donation. Fraud was found in less than 5% of the sample but required an outsized amount of staff time to detect and manage.
